# Risk of Adverse Events in Cancer Patients Receiving Nivolumab With Ipilimumab: A Meta-Analysis

**DOI:** 10.3389/fonc.2022.877434

**Published:** 2022-06-23

**Authors:** Xin Zhao, Fengwei Gao, Jie Yang, Hua Fan, Qingyun Xie, Kangyi Jiang, Jie Gong, Benjian Gao, Qian Yang, Zehua Lei

**Affiliations:** ^1^ Department of Hepatobiliary Surgery, The People’s Hospital of Leshan, Leshan, China; ^2^ Department of Medical Oncology, The People’s Hospital of Leshan, Leshan, China

**Keywords:** immune checkpoint inhibitors (ICI), ipilimumab, adverse events, nivolumab, meta-analysis

## Abstract

**Background:**

Combining two immune checkpoint inhibitors (ICIs) instead of using one can effectively improve the prognosis of advanced malignant tumors. At present, ipilimumab alongside nivolumab is the most widely used combinatorial regimen of ICIs. However, the risk of treatment-related adverse events is higher in combinatorial regimens than in single-drug regimens. Thus, this study aimed to evaluate the risks of common adverse events associated with the combinatorial regimen of ipilimumab and nivolumab by using meta-analysis.

**Methods:**

We searched Pubmed, Medline, EMBASE, and Cochrane Library for reports published by 30 September 2021. A randomized controlled study was developed and analyzed using the statistical software R to determine the efficacy of the combinatorial treatment. Risk estimates (hazard ratios, RR) and 95% confidence intervals for various common serious adverse events were used.

**Results:**

A total of 23 randomized control trials (n = 3970 patients) were included. Our meta-analysis indicated the risks of adverse events of any grade and grade ≥ 3 as 90.42% (95%CI: 85.91% ~ 94.18%) and 46.46% (95%CI: 39.37% ~ 53.69%), respectively; the risks of treatment-related death and adverse events leading to discontinuation were estimated at 0.42% (95% CI, 0.18% ~ 0.72%) and 19.11% (95% CI, 14.99% ~ 24.38%), respectively. Classification of 19 common adverse events. The top 5 grade 1-2 adverse events were found to be fatigue (30.92%, 95% CI: 24.59% ~ 37.62%), pruritus (26.05%, 95%CI: 22.29%~29.99%), diarrhea (23.58%, 95% CI: 20.62% ~ 26.96%), rash (19.90%, 95%CI: 15.75% ~ 25.15%), and nausea (17.19%, 95% CI:13.7% ~ 21.57%). The top 5 grade ≥ 3 adverse events were identified as increased alanine aminotransferase(8.12%, 95% CI: 5.90%~10.65%), increased lipase(7.62%, 95% CI: 4.88% ~ 10.89%), and colitis (6.39%, 95%CI: 3.98% ~ 10.25%), increased aspartate aminotransferase (6.30%, 95% CI: 4.61% ~ 8.22%), and diarrhea(5.72%, 95%CI: 3.50% ~ 8.44%). Subgroup analysis revealed some differences in the adverse events between the N1-I3 and N3-I1 subgroups and between subgroups of different cancer types.

**Conclusion:**

This study summarized the risks of common adverse events in the co-treatment of malignant-tumor patients with ipilimumab and nivolumab and identified the impacts of various initial administration schemes on the risks of such events, thereby providing an important reference for the toxicity of co-treatment with ipilimumab and nivolumab.

**Systematic Review Registration:**

https://www.crd.york.ac.uk/prospero/, identifier: CRD42020181350.

## Introduction

According to the estimates of the World Cancer Center and the American Cancer Center, there were 9 million cancer-related deaths worldwide in 2020 and 600000 in the United States in 2021 (1, 2). Surgical treatment, radiotherapy, chemotherapy, and targeted drug treatment are the common treatment strategies for malignant tumors. However, these approaches have limited effects on some advanced malignant tumors. The in-depth studies on immune checkpoint inhibitors (ICIs) in recent years have provided a good prospect for the treatment of advanced malignant tumors (3, 4). ICIs are monoclonal antibodies that can activate the immune system to enhance antitumor immunity. The results of many large-scale multicenter randomized control trials (RCTs) have shown that immunotherapy can effectively prolong the survival of patients with advanced malignant tumors, and some immunotherapeutic drugs have become the first-line antitumor therapeutics (5, 6). At present, common ICIs include ipilimumab, tremelimumab, nivolumab, pembrolizumab, atezolizumab, and durvalumab. Studies have shown that the efficacy of single-drug therapies is limited, and thus combinatorial immunotherapy is gradually becoming the focus of cancer research worldwide (7, 8). Multi-phase clinical trials on combinatorial therapies involving immune-targeted therapy, chemoradiotherapy, or two ICIs have yielded gratifying results. Ipilimumab alongside nivolumab is the most common combination of two ICIs in cancer treatment and has been successfully applied to malignant tumors, such as advanced malignant melanoma and lung and kidney cancers (9). Combinatorial immunotherapy can have a good curative effect but lacks selectivity and specificity, inhibits both normal and abnormal immune responses, and is often accompanied by some adverse events. Although several meta-analysis studies have reported the risk of adverse events associated with some combinatorial immunotherapy regimens, the plausible combinations of immunotherapy drugs are extensive, and no such study has been reported on the combinatorial use of ipilimumab and nivolumab (10–12). Therefore, this study aimed to evaluate the risk of various common adverse events associated with the combinatorial use of ipilimumab and nivolumab, thereby providing an evidence-based basis for the management of such events in the clinic.

## Materials and Methods

### Literature Review and Study Identification

This systematic review and meta-analysis was conducted according to the Preferred Reporting Items for Systematic Reviews and Meta Analyses (PRISMA) and Assessing the Methodological Quality of Systematic Reviews (AMSTAR) guidelines. This study was registered on Prospero (Registration number: CRD42020181350). Two independent researchers searched the PubMed, EMBASE, Cochrane Library, and MEDLINE databases for the relevant literature published between the beginning of database construction and September 30, 2021, and extracted the relevant data. The search keywords were “nivolumab”, “ipilimumab”, “CTLA-4”, and “PD-1”. We also manually checked the supplementary materials and list of references in each retrieved article to further identify any potential relevant RCT and searched the websites of the relevant regulatory agencies in the United States and Europe [The Federal Drug Administration and European Drug Administration, respectively]. We reported the basis of this systematic review and meta-analysis in accordance with Cochrane’s recommendations on preferred reporting items for systematic review and meta-analysis.

### Article Selection

We included only phase I–IV RCTs on ipilimumab and nivolumab combinatorial therapy of patients with malignant tumors. We excluded non-randomized trials, and studies with malignant-tumor patients, additional regimens (e.g., radiotherapy, chemotherapy, and targeted therapy), incomplete data on adverse events, or high bias in risk assessment. If two reports corresponded to the same study of a research group, we included only the most complete and up-to-date study. Two reviewers independently screened all titles, abstracts, and full texts to assess whether the corresponding studies qualified. Any disagreement among these reviewers was judged by a third researcher and finally resolved by consensus.

### Data Extraction and Quality Assessment

Two researchers repeatedly extracted data according to the preset extraction table. Any inconsistency in extracted data between the two researchers was resolved *via* discussion with a third researcher. The extracted data included the study registration number, first author, publication year, trial stage, tumor type, number of cases, treatment scheme, and initial dose scheme of each included study. We defined adverse events ≥ 10 literatures as common adverse events. The extracted analysis data included the total number of adverse events of any grade, grade 1-2, and grade ≥ 3, as well as adverse events leading to drug withdrawal and death. We evaluated the potential bias risk of each included RCT by using the bias-risk assessment tool of the Cochrane Center.

### Statistical Analysis

The meta-analysis was performed using R statistical soft-ware (packages metafor and meta, R Foundation) (13). We used the R software to calculate the risk ratio and 95% confidence interval of each outcome index and to perform logarithmic, logit, anti-sinusoidal, and double anti-sinusoidal transformations on the analysis data to test the normal distribution of each transformation. For each set of data, we finally select the set of 4 transformed data that is closest to the normal distribution for meta-analysis. If there was significant heterogeneity (I^2^> 50%), the random effect model was selected, otherwise, the fixed-effect model was used. For subgroup analysis, we sub-grouped the patients into N1–I3 subgroup (nivolumab 1 mg/kg + ipilimumab 3 mg/kg) and N3–I1 subgroup (nivolumab 3 mg/kg + ipilimumab 1 mg/kg), according to the different initial administration schemes. Finally, we used the Graphpad (version 9.2) software to draw the classification summary of results and used Egger’s test to evaluate publication bias. The significance level of the bilateral test was set at p < 0.05.

## Results

### Eligible Studies and Characteristics

**Figure 1:**
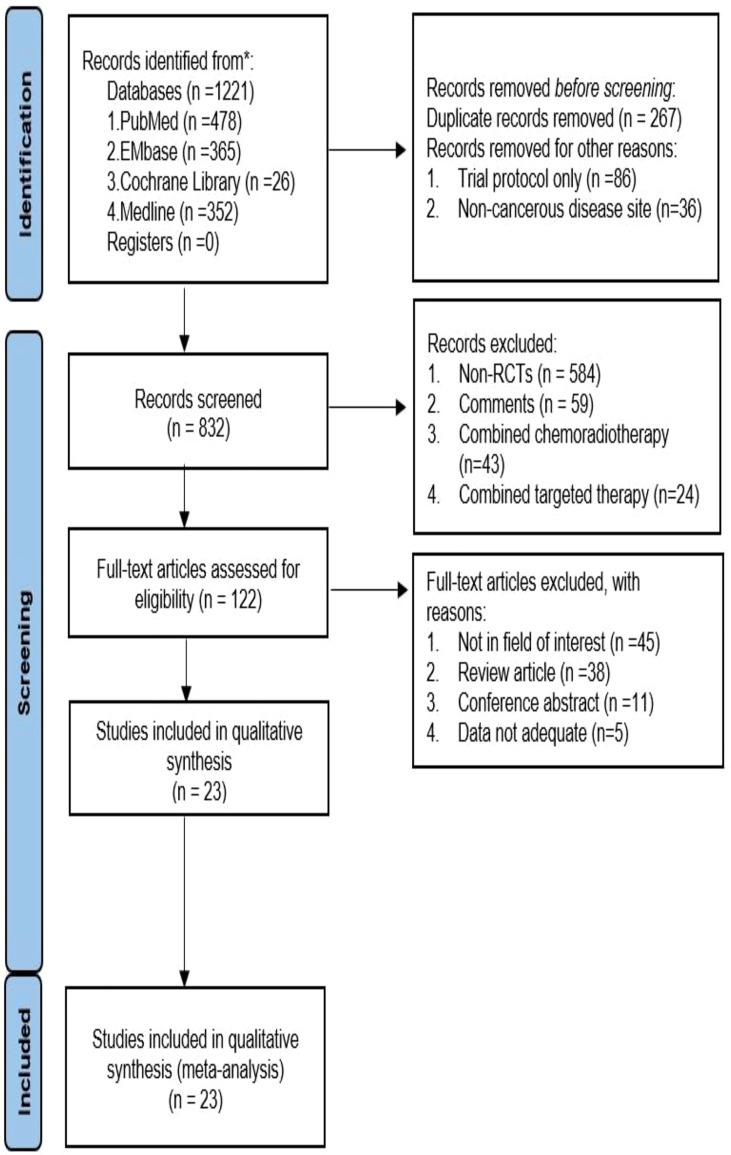
PRISMA Flow diagram.

We initially retrieved 1221 studies following the set retrieval strategy. Duplicate records were subsequently eliminated, leaving 832 studies after excluding trial protocol and non-cancerous disease site. Another 710 studies were excluded after reading the title and abstract, Including 584 non-randomized controlled studies(Non-RCTs), 59 were Comments, 43 were Combined chemoradiotherapy, 24 were Combined targeted therapy. Full-text reading of the 122 studies led to the elimination of 99 articles. Amongst the 99 studies, 45 were not in the field of interest, 38 were review articles, 11 were conference abstracts, and 5 had insufficient data. The remaining 23 studies were included for meta-analysis (14–36), which included 32 single arms (see **Figure 1** and **Table 1**) and a total of 3970 patients with malignant tumors. The tumor types included malignant melanoma, non-small cell lung cancer, advanced renal cell carcinoma, malignant pleural mesothelioma, malignant sarcoma, esophageal gastric junction cancer, colorectal cancer, malignant glioma, and urothelial, ovarian, and hepatocellular carcinomas. According to the number of reports, we analyzed 19 common adverse events, namely diet, pruritus, diarrhea, rash, nausea, hyperthyroidism, hyperthyroidism, discredited appetite, pyrexia, headache, maculopapular rash, pneumonitis, adrenal insufficiency, colitis, vomiting, and increased aspartate aminotransferase (AST), increased alanine aminotransferase (ALT), increased amylase, and increased lipase.

**Table 1:** Characteristics of the included studies.

NCT number	Author	Year	Phase	No. of patients	Median age (years)	Male (%)	Cancer type	Follow-up (Median months)	Dose (I+N)
NCT01927419	Hodi	2016	II	94	NA	NA	Melanoma	24.5	3mg/kg+1mg/kg
NCT01454102	Hellmann	2017	I	77	68 (58∼73)	25.00%	NCLC	12.8	3mg/kg/+1mg/kg
					62 (57∼73)	38.71%			3mg/kg/+1mg/kg
NCT01472081	Hammers	2017	I	47	54 (26∼68)	79.63%	RCC	22.3	1mg/kg+3mg/kg
NCT01472081	Hammers	2017	I	47	56 (20∼76)	64.29%	RCC		3mg/kg+1mg/kg
NCT01844505	Wolchok	2017	III	313	NA	NA	Melanoma	36	1mg/kg+3mg/kg
NCT02437279	Blank	2018	I	20	54 (40∼58)	12.96%	Melanoma	25.6	3mg/kg+1mg/kg
NCT02500797	D’Angelo	2018	II	42	57 (27∼81)	19.00%	Sarcoma	13.6	1mg/kg+3mg/kg
NCT02374242	Long	2018	II	35	59 (53–68)	83%	Melanoma	17	3mg/kg+1mg/kg
NCT01844505	Hodi	2018	III	313	NA	NA	Melanoma	48	3mg/kg+1mg/kg
NCT02320058	Tawbi	2018	II	94	59 (22∼81)	65.00%	Melanoma	14	3mg/kg+1mg/kg
NCT01928394	Janjigian	2018	I/II	49	53 (22∼77)	34.00%	Esophagogastric cancer	24	3mg/kg+1mg/kg
NCT01928394	Janjigian	2018	I/II	52	58 (19∼81)	45.00%	Esophagogastric cancer	22	1mg/kg+3mg/kg
NCT02060188	Overman	2018	II	119	58 (21∼88)	70.00%	Colorectal Cancer	13.4	1mg/kg+3mg/kg
NCT02017717	Omuro	2018	I	10	57 (37∼68)	6.00%	Glioblastoma	NA	3mg/kg+1mg/kg
NCT02017717	Omuro	2018	I	20	60 (27∼73)	14.00%	Glioblastoma		1mg/kg+3mg/kg
NCT02477826	Hellmann	2019	III	576	64 (26∼87)	67.40%	NCLC	24	1mg/kg/+3mg/kg
NCT02714218	Lebbé	2019	III/IV	180	58.5 (19∼85)	58.30%	Melanoma	12	1mg/kg+3mg/kg
NCT02714218	Lebbé	2019	III/IV	178	58.5 (26∼85)	56.70%	Melanoma		3mg/kg+1mg/kg
NCT02659059	Ready	2019	II	288	65 (39∼91)	49.30%	NCLC	6	1mg/kg+3mg/kg
NCT02231749	Motzer	2019	III	547	NA	NA	RCC	32.4	1mg/kg+3mg/kg
NCT02977052	Rozeman	2019	II	30	64 (18∼79)	19.00%	Melanoma	18	3mg/kg+1mg/kg
NCT02977052	Rozeman	2019	II	30	54 (31∼74)	14.00%	Melanoma		1mg/kg+3mg/kg
NCT02716272	Scherpereel	2019	II	61	71.2 (48.1∼88.1)	53.00%	Pleural mesothelioma	20.1	1mg/kg+3mg/kg
NCT01928394	Sharma	2019	I/II	104	63 (39∼83)	77.90%	Urothelial carcinoma	38.8	1mg/kg+3mg/kg
NCT01928394	Sharma	2019	I/II	92	64 (38∼83)	80.40%	Urothelial carcinoma	7.9	3mg/kg+1mg/kg
NCT02523313	Zimmer	2020	II	55	52 (45∼59)	55.00%	Melanoma	12.4	3mg/kg+1mg/kg
NCT01658878	Yau	2020	I/II	49	NA	NA	HCC	30.7	3mg/kg+1mg/kg
NCT01658878	Yau	2020	I/II	97			HCC		1mg/kg+3mg/kg
NCT02498600	Zamarin	2020	II	51	62 (38∼92)	NA	Ovarian Cancer	33	1mg/kg+3mg/kg
NCT02899299	Baas	2021	III	300	69 (65∼75)	77.00%	Malignant pleural mesothelioma	29.7	1mg/kg+3mg/kg

NCLC, Non-small-cell lung cancer; RCC, Renal cell carcinoma; HCC, Hepatocellular Carcinoma; NA, Nae.

### Incidence of any adverse events and risk ratio of grade 3 or higher adverse events

**Figure 2:**
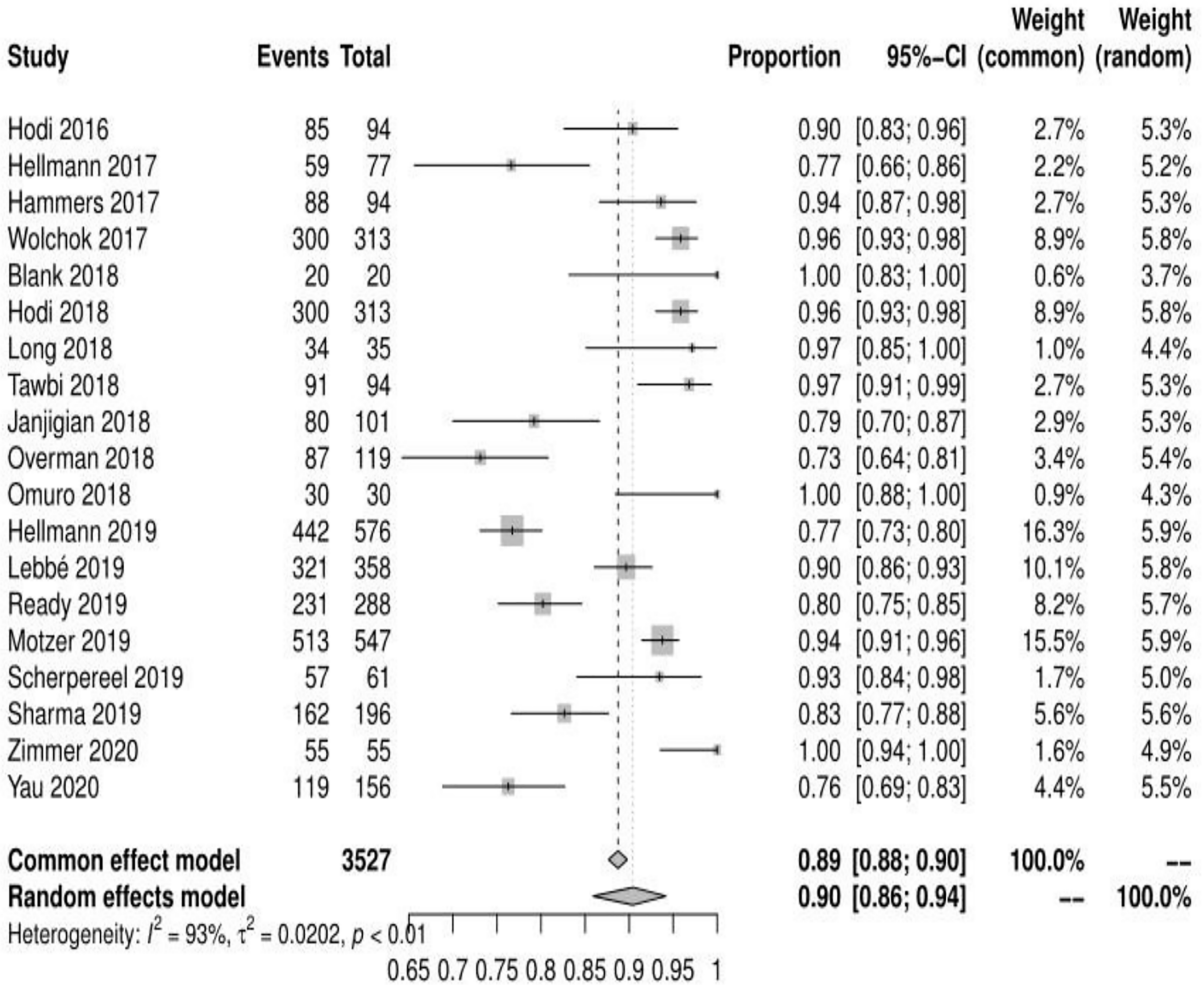
Forest plot of any adverse events.

**Figure 3:**
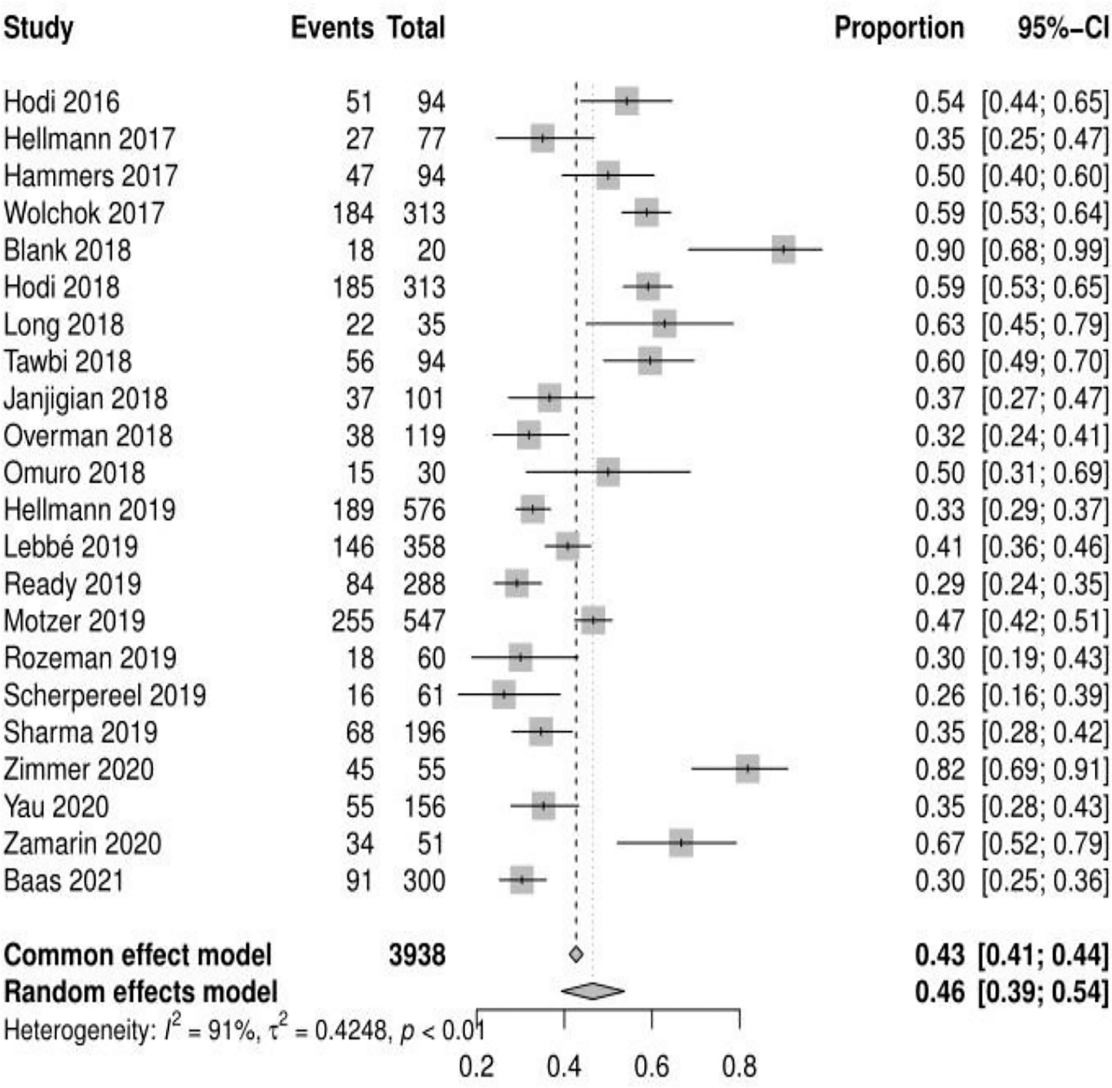
Forest plot of grade 3 or higher adverse events.

Of the 23 studies analyzed, 19 reported adverse events of any grade, the mean incidence of any adverse events was 90.42% (95% CI, 85.91% ~ 94.18%, I^2 =^ 93%) **(**see **Figure 2** **)**. 22 reported adverse events of grade ≥ 3, and the mean incidence of grade 3 or higher adverse events was 46.46% (95% CI, 39.37% ~ 53.69%, I^2 =^ 91%) **(**see **Figure 3** **)**. Subgroup analysis revealed that the mean incidence of any adverse event and that of a grade ≥ 3 adverse event were 94.53% (95% CI, 91.18% ~ 97.21%, I^2 =^ 71%) and 55.29% (95% CI, 46.73% ~ 63.86%, I^2 =^ 85%) in the N1–I3 subgroup **(**see **Supplementary Material Figure S1**, **S2** **)**, respectively, and 84.91% (95% CI, 80.02% ~ 90.10%, I^2 =^ 90%) and 36.72% (95% CI, 30.51% ~ 43.39%, I^2 =^ 81%) in the N3–I1 subgroup **(**see **Supplementary Material Figure S3**, **S4** **)**.

### Incidence of Treatment-Related Deaths and Treatment−Related Adverse Event Leading to Discontinuation

**Figure 4:**
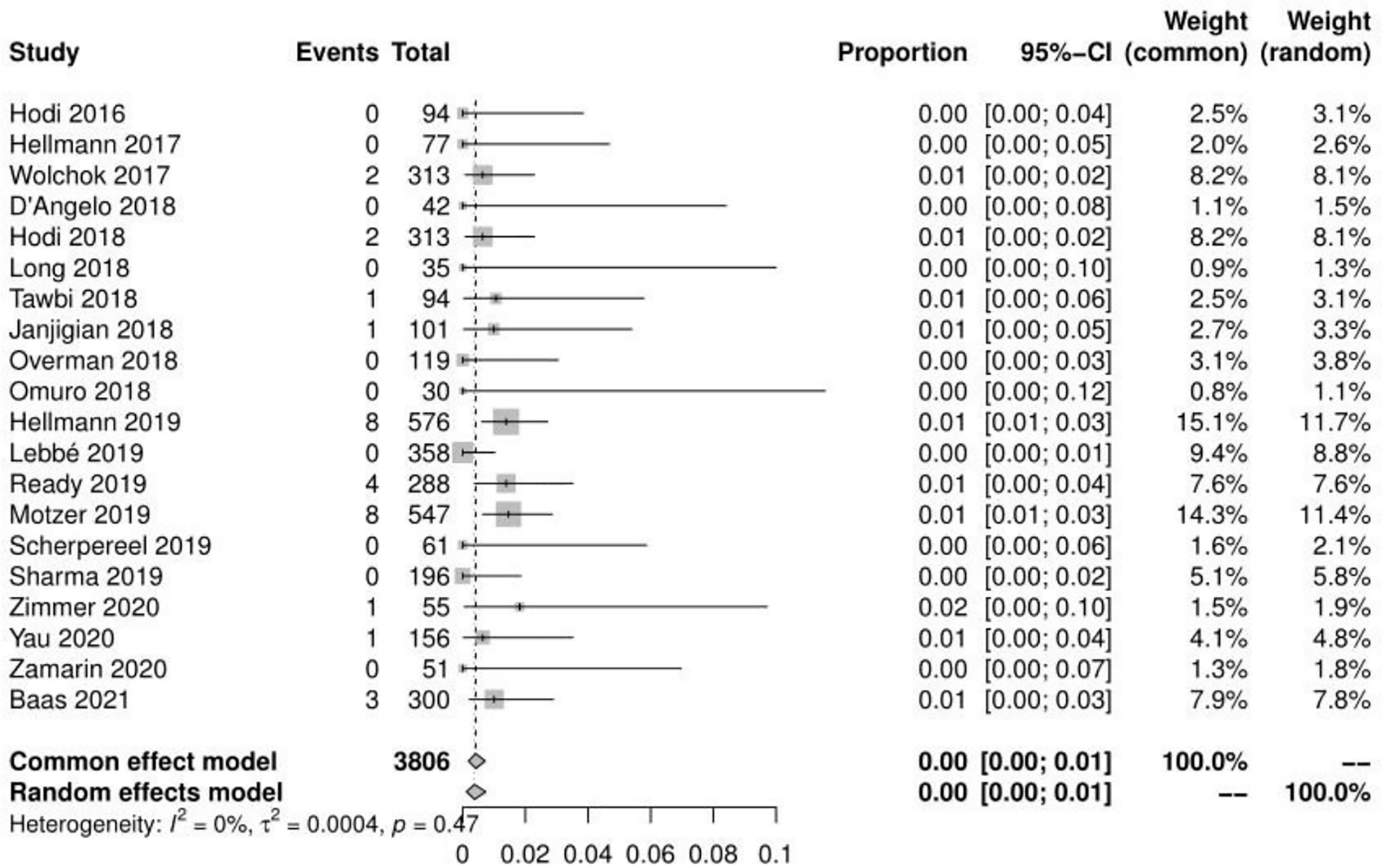
Forest plot of treatment-related deaths.

**Figure 5:**
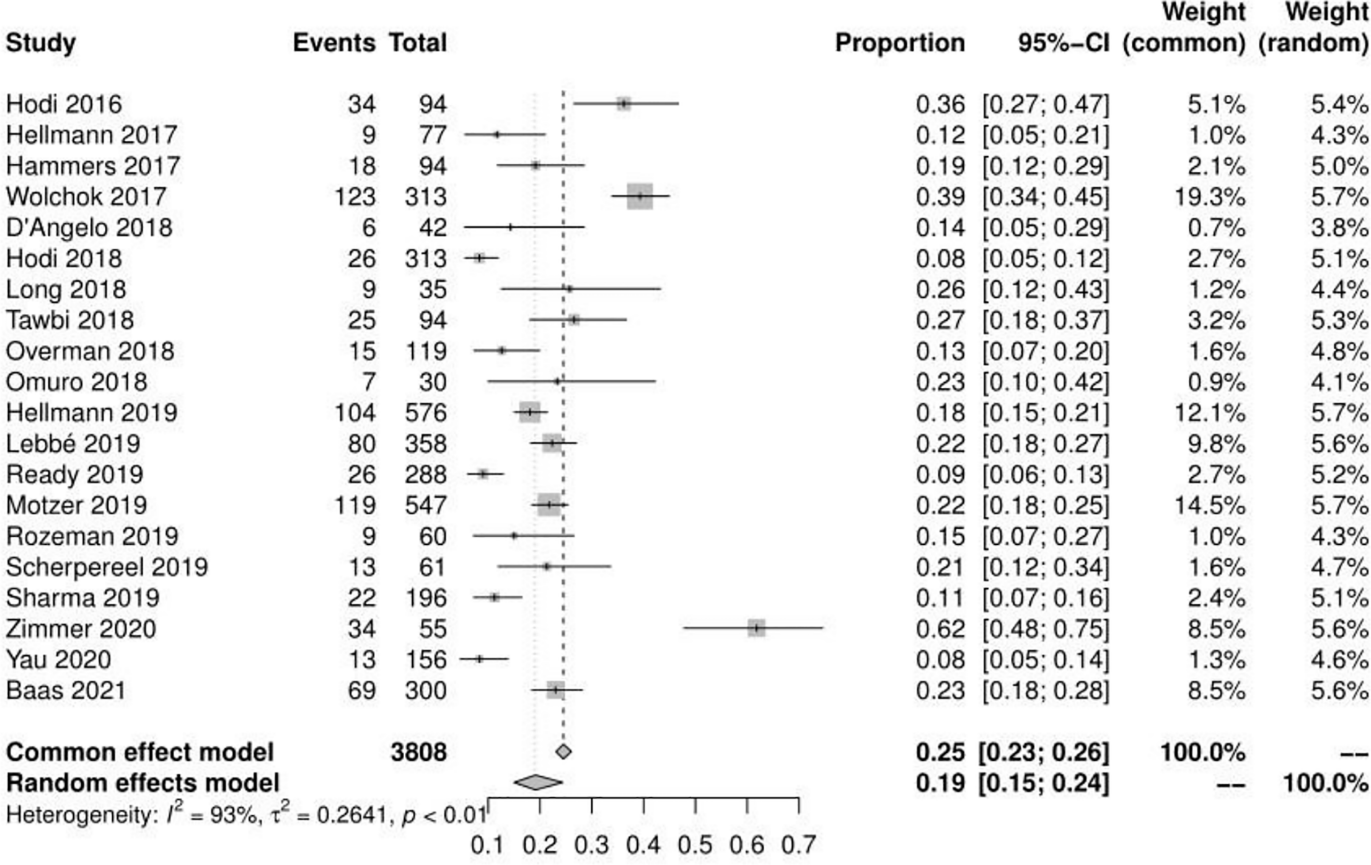
Forest plot of adverse events leading to discontinuation.

Of the 23 studies, 20 reported a total of 31 treatment-related deaths, with a mean incidence of 0.42%(95% CI, 0.18% ~ 0.72%, I^2 =^ 0%) (see **Figure 4**). 20 reported the number of adverse events leading to discontinuation, with a mean incidence of 19.11% (95% CI, 14.99% ~ 24.38%, I^2 =^ 93%) (see **Figure 5**). The mean incidence of treatment-related death and that of a treatment-related adverse event leading to discontinuation were 0.06% (95% CI, 0.00% ~ 0.44%, I^2 =^ 0%) and 27.51% (95% CI, 21.45% ~ 35.29%, I^2 =^ 83%) in the N1–I3 subgroup**(**see **Supplementary Material Figure S5**-**S6** **)**, respectively, and 0.43% (95% CI, 0.14% ~ 0.83%, I^2 =^ 0%) and 14.65% (95% CI, 11.54% ~ 18.04%, I^2 =^ 75%) in the N3–I1 subgroup**(**see **Supplementary Material Figure S7**-**S8** **)**.

### Risk Ratio of Grade 1 and 2 Adverse Events

**Figure 6:**
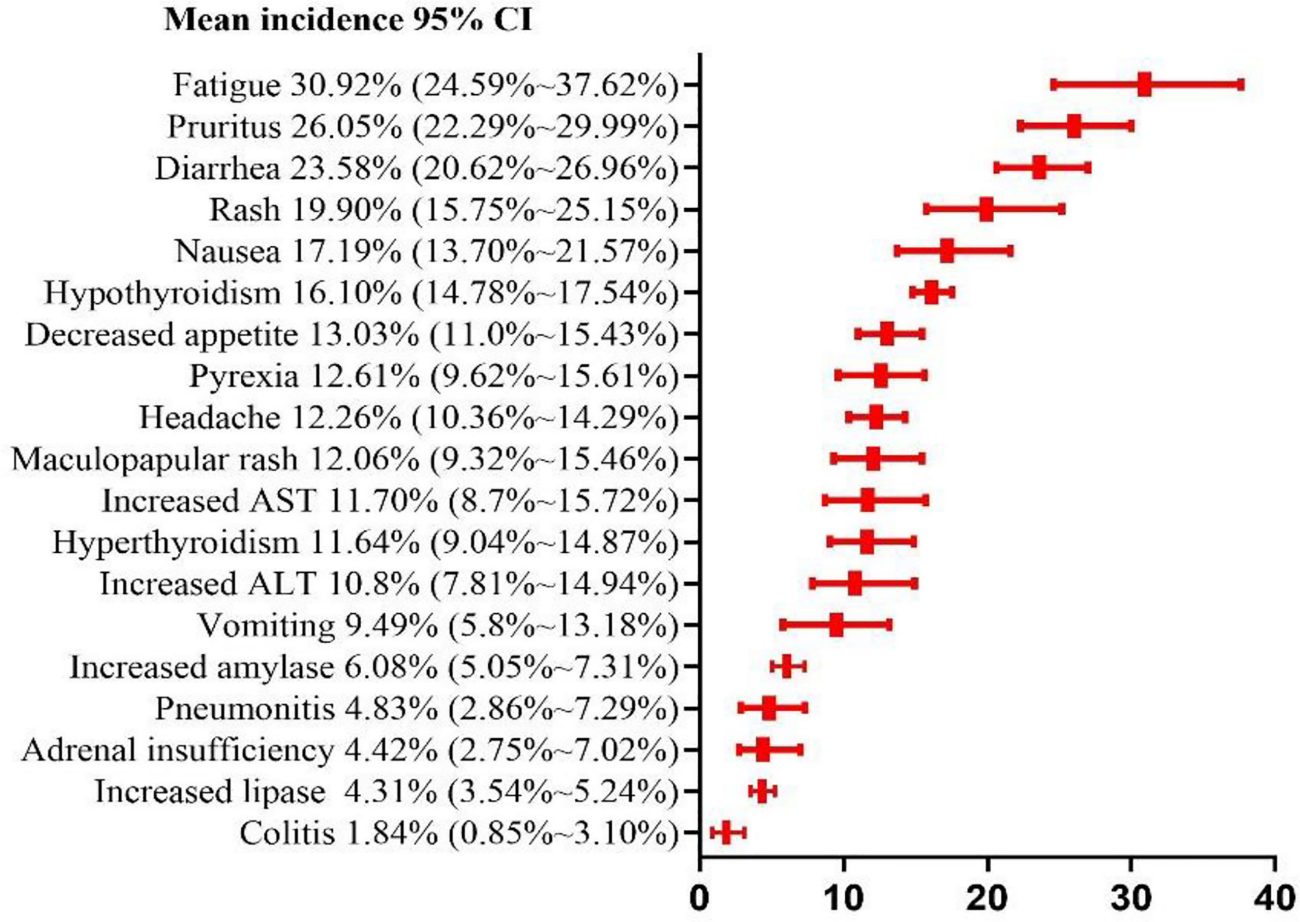
Risk ratio of grade 1 and 2 adverse events.

Among the 19 common adverse events analyzed, the risk of grade 1–2 adverse events was > 10%. The top 5 risks were fatigue(30.92%, 95% CI: 24.59% ~ 37.62%, I^2 =^ 93%), pruritus (26.05%, 95% CI: 22.29% ~ 29.99%, I^2 =^ 82%), diarrhea(23.58%, 95% CI: 20.62% ~ 26.96%, I^2 =^ 88%), rash(19.90%, 95% CI: 15.75% ~ 25.15%, I^2 =^ 88%), nausea (17.19%, 95% CI: 13.7% ~ 21.57%, I^2 =^ 86%), the risks of other common adverse events are presented in **Figure 6**. Fatigue, pruritus, diarrhea, and rash were also among the top 5 risks in the N1–I3 and N3–I1 subgroups, which additionally included nausea and hypothyroidism, respectively. The risks of other common adverse events in each subgroup are presented in **Table 2**.

**Table 2:** Risk ratio of grade 1 and 2 adverse events for N3-I1and N1-I3 subgroup.

Subgroup	Mean incidence 95%CI	
	N3-I1	N1-I3
Fatigue	23.01% (17.63%~29.43%)	38.67% (31.17%~47.97%)
Pruritus	22.34% (18.72%~25.96%)	30% (24.06%~35.95%)
Diarrhea	18.95% (17.41%~20.54%)	27.18% (22.92%~31.64%)
Rash	16.43% (13.77%~19.61%)	26.18% (18.66%~34.47%)
Hypothyroidism	14.46% (11.49%~18.21%)	16.77% (14.75%~18.79%)
Nausea	13.22% (9.82%~17.04%)	20.45% (15.10%~26.39%)
Headache	12.67% (5.80%~21.40%)	13.54% (11.44%~15.96%)
Pyrexia	11.21% (8.86%~14.19%)	13.59% (9.53%~17.64%)
Decreased appetite	10.96% (9.56%~12.44%)	15.74% (13.83%~17.87%)
Hyperthyroidism	8.45% (5.24%~12.34%)	13.92% (9.32%~10.29%)
Maculopapular rash	8.20% (6.59%~9.80%)	15.04% (10.79%~20.58%)
Increased ALT	7.94% (4.06%~12.97%)	11.61% (8.77%~14.75%)
Increased AST	7.07% (3.69%~11.44%)	14.86% (11.67%~18.93%)
Increased amylase	5.75% (2.23%~10.49%)	6.47% (5.17%~8.07%)
Vomiting	5.12% (1.95%~9.37%)	13.06% (11.15%~15.24%)
Adrenal insufficiency	5.09% (2.08%~9.33%)	3.68% (1.05%~7.83%)
Increased lipase	3.24% (2.12%~4.54%)	5.16% (3.52%~7.5%)
Pneumonitis	3.22% (1.94%~4.73%)	6.75% (4.74%~8.76%)
Colitis	0.62% (0.11%~1.39%)	1.74% (0.27%~4%)

### Risk Ratio of Grade 3 or Higher Adverse Events

**Figure 7:**
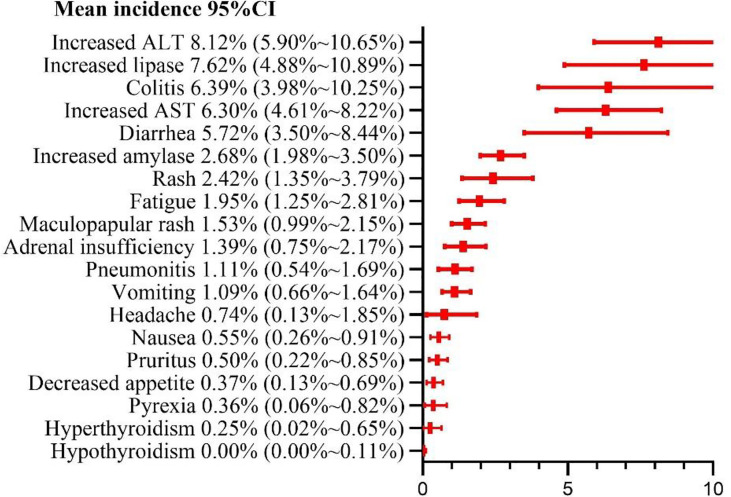
Risk ratio of grade 3 or higher adverse events.

Among the 19 common adverse events, the risk of a grade ≥ 3 adverse event was > 5%. The top 5 risks were increased ALT (8.12%, 95% CI: 5.90% ~ 10.65%, I^2 =^ 73%), increased lipase (7.62%, 95% CI: 4.88% ~ 10.89%, I^2 =^ 77%), colitis (6.39%, 95% CI: 3.98% ~ 10.25%, I^2 =^ 80%), increased AST (6.30%, 95% CI: 4.61% ~ 8.22%, I^2 =^ 68%), diarrhea (5.72%, 95% CI: 3.50% ~ 8.44%, I^2 =^ 83%), the risks of other common adverse events are presented in **Figure 7**. Sub-group analysis yielded the same adverse events as the top 5 risks in both N1–I3 and N3–I1 subgroups. The risks of other common adverse events are presented in **Figure 7** and **Table 3**.

**Table 3:** Risk ratio of grade 3 and higher adverse events for N3-I1 and N1-I3 subgroup.

Subgroup	Mean incidence 95%CI	
	N3-I1	N1-I3
Increased Lipase	6.14% (2.46%~11.32%)	9.13% (5.60%~13.41%)
Increased ALT	3.99% (2.21%~6.27%)	11.02% (7.9%~14.58%)
Increased AST	3.85% (2.22%~5.91%)	7.96% (5.44%~10.91%)
Colitis	3.31% (0.83%~7.35%)	7.86% (4.58%~11.13%)
Diarrhea	2.77% (2.17%~3.45%)	9.17% (6.06%~13.65%)
Rash	1.94% (0.55%~1.89%)	2.32% (1.44%~3.34%)
Increased Amylase	1.87% (0.21%~5.11%)	3.50% (2.57%~4.76%)
Fatigue	1.30% (0.85%~1.83%)	2.21% (1.37%~3.20%)
Adrenal insufficiency	1.27% (0.45%~2.35%)	1.14% (0.29%~2.36%)
Pneumonitis	0.64% (0.07%~1.55%)	0.51% (0.00%~1.56%)
Maculopapular rash	0.54% (0.09%~1.24%)	1.96% (1.05%~3.07%)
Vomiting	0.46% (0.00%~2.39%)	1.21% (0.49%~2.15%)
Headache	0.26% (0.00%~4.27%)	0.20% (0.00%~0.85%)
Pruritus	0.21% (0.01%~0.60%)	0.43% (0.05%~1.05%)
Nausea	0.10% (0.00%~0.41%)	1.08% (0.46%~1.87%)
Decreased appetite	0.04% (0.00%~0.31%)	0.74% (0.34%~1.30%)
Hyperthyroidism	0.00% (0.00%~0.12%)	0.49% (0.06%~1.17%)
Hypothyroidism	0.00% (0.00%~0.08%)	0.00% (0.00%~0.13%)
Pyrexia	0.00% (0.00%~0.39%)	0.29% (0.00%~0.91%)

### Subgroup Analysis of the Incidence of Adverse Events Based on Cancer Type

Based on the risk of any adverse events (**Supplementary Material Figure S9**), melanoma had the highest risk (95.87%, 95% CI: 92.93% ~ 98.12%, I2 = 72.4%), while colorectal cancer had the lowest risk (73.11%, 95% CI: 64.75% ~ 80.73%, I2 = 0%). Similarly, based on the risk of grade 3 and higher adverse events (**Supplementary Material Figure S10**), melanoma had the highest risk (58.13%, 95% CI: 47.67% ~ 70.88%, I2 = 92.3%), while pleural mesothelioma had the lowest risk (26.23%, 95% CI: 17.22% ~ 39.95%, I2 = 0%). Melanoma also had the highest risk (25.83%, 95% CI: 16.79) % ~ 39.75%, I2 = 94.2%) based on risk of any adverse event leading to discontinuation (**Supplementary Material Figure S11**), while glioblastoma had the lowest risk (23.33%, 95% CI: 12.20% ~ 44.64%, I2 = 0%). In contrast, glioblastoma had the highest risk (1.64%, 95% CI: 0.10% ~ 25.62%, I2 = 0%) based on the risk of treatment-related deaths (**Supplementary Material Figure S12**), while urothelial carcinoma had the lowest risk (0.25%, 95% CI: 0.02% ~ 4.05%, I2 = 0%).

## Discussion

ICIs are monoclonal antibodies against regulatory immune checkpoint factors that inhibit T cell activation. These antibodies promote immune-mediated tumor-cell clearance by enhancing T cell-mediated anti-tumor immunity. At present, their targets mainly include CTLA-4 and PD-1/PD-L1, CTLA-4 regulates the activation of T cells by preventing the generation of T cell inhibitory signals (37, 38). let’s first talk about the five common adverse events, and then describe whether the risk of grade ≥ 3 is > 5%, so as to promote the further proliferation of T cells, thereby achieving the anti-tumor effect of PD-1. PD-L1 inhibits the signal transduction by blocking the interaction between T cells and antigen-presenting cells, promotes the proliferation of activated T cells, and then kills tumor cells (39, 40). However, activation of the immune system also impairs the immune homeostasis in non-tumor tissues, resulting in a series of adverse reactions, mainly involving the skin, gastrointestinal tract, liver, lung, and endocrine glands (41, 42). In recent years, combinatorial immunotherapy has gradually become a research hotspot, and its antitumor effectiveness has been confirmed by multiple studies. However, combination of drugs seems to increase the risk of adverse events. The results of the meta-analyses by Yang et al. and Chen et al (43, 44). have shown that the antitumor effect of nivolumab and ipilimumab co-treatment was better than that of nivolumab or ipilimumab alone. The results of the meta-analysis by Xing et al. have shown that the risk of adverse events related to nivolumab and ipilimumab co-treatment was higher than that of the single-drug use (45). To the best of our knowledge, the study presented here is the largest and most comprehensive meta-analysis to evaluate the common adverse events of nivolumab and ipilimumab combination. The study by Xing et al. analyzed fewer studies than this study and did not include subgroup analysis of the initial medication regimen.

From the perspective of patient consultation, several results of this meta-analysis are crucial. Our results showed that approximately 9 of the 10 patients treated with nivolumab alongside ipilimumab had at least one adverse event, and 5 of the 10 patients had at least one grade ≥ 3 adverse event. Among them, fatigue was the most common mild adverse event (30.92%), and increased ALT level was the most common grade ≥ 3 adverse event (8.12%). Patients should also be informed that pruritus, diarrhea, and rash are also common adverse events but are remotely likely to manifest as serious complications. The fatality rate of any of these adverse events was very low (0.5%). The risk of drug withdrawal due to an adverse event was estimated at 42%. Approximately 1 of the 5 patients discontinued the treatment because of an adverse event.

In this meta-analysis, fast, headache, decreased appetite, and pyrexia were found to be subjective symptoms. Of the remaining adverse events, diarrhea, nausea, vomiting, colitis, and increased AST, ALT, amylase, and lipase levels are related to the digestive system; rash, pruritus, and maculopapular rash are skin-related; hyperthyroidism and adult insufficiency are endocrine-related; and pneumonitis is mainly related to the respiratory system. Regarding the grade 1–2 adverse events, 13 had risks of > 10%, and 15 had > 5%. Fatigue (30.92%) had the highest risk, which was higher than the risk of adverse events reported in PD-1 (18.7%) and PD-L1 (26%) meta-analysis studies by Wang et al (42). Grade 1–2 adverse events often do not have serious consequences for patients but increase patient discomfort and weaken the eagerness of the patient for the treatment. Some grade 1–2 adverse events often develop into grade ≥ 3 adverse events, such as colitis and pneumonitis, if not managed timely. We found that 5 grade ≥ 3 events had risks of > 5%, and 12 had > 1%. Among such events, increased ALT (8.12%) level was the most common. Increased ALT and AST levels are symptoms of hepatitis; increased lipase and amylase levels are symptoms of pancreatitis; hyperthyroidism and hyperthyroidism are symptoms of thyroiditis; diarrhea is a symptom of colitis. If autoimmune diseases are not identified early, they often cause severe health problems and can even be fatal. Pneumonia is the most common cause of treatment-related deaths in patients treated with immunosuppressants, and we estimated the incidence at 1.5% and 11%. In addition, our results show that the types of adverse events in the digestive system and their risks are significantly higher than those in other systems. Therefore, it is necessary to monitor the digestive system of the patients under treatment for such events to prevent development of severe problems in the digestive system.

In our study, we sub-grouped the patients according to their initial dosing regimen, namely N1–I3 and N3–I1 subgroups, and performed subgroup analysis. The N3–I1 subgroup had higher risks of adverse events of any grade, grade 1–2, and grade ≥ 3 (both with and without classification) than the N1–I3 subgroup, consistent with the results of the meta-analysis by Xu et al (46). The risk of adverse events of any grade was not classified, and nearly 10% (94.53% vs. 84.51%) of the N3–I1 subgroup had a higher risk than the N1–I3 subgroup, whereas nearly 20% (55.29% vs. 36.72%) of the N3–I1 subgroup had a higher risk of adverse events of grade ≥ 3 than the N1–I3 subgroup. Regarding the risk of classified grade 1 and grade 2 common adverse events, the most common risk in both N1–I3 and N3–I1 subgroups was fatigue (38.67% and 23.01%, respectively), whereas, regarding the classified grade ≥ 3 common adverse events, the most common risks in the N1–I3 and N3–I1 subgroups were increased ALT (11.02%) and lipase (6.14%) levels, respectively. Therefore, initial medication schemes have a certain impact on the occurrence of adverse events. When deciding on the medication scheme, treatment effectiveness and cost should also be considered in addition to treatment safety. We should be more cautious about the impact of different initial medication regimens of N3-I1 and N1-I3 on adverse events, because we did not consider the impact of treatment period and sequence.

Our preliminary analysis of the incidence of adverse events in different types of tumors further revealed that melanoma had a higher overall risk of adverse events. However, the differences between different cancer types were not significant. A meta-analysis of PD-1 and PD-L1 by Wang et al. revealed a similar average incidence of adverse events across various cancer types (42). However, this conclusion could not be fully explained in our study, possibly because of the small sample size of some tumor types. Some studies postulate that there are certain differences in the risk of adverse events for different types of tumors (47, 48). Based on the dose subgroup analysis for different cancer types, choosing the best drug regimen can prevent the occurrence of some adverse events to a certain extent. Taking timely intervention measures to the occurrence of common adverse events can further reduce the occurrence of serious adverse events and deaths.

Nonetheless, this study was limited by several factors, some with high heterogeneity (I2> 90%). We did not conduct subgroup analysis by cancer type for different types of adverse events. We also did not further analyze race, age, gender, and smoking history, amongst other demographic and clinical factors, which may have led to deviations in the analysis results. The length of treatment cycles may also have impacted the results despite performing subgroup analyses based on different initial doses. The different follow-up times for each study may have also biased the results.

In conclusion, this study estimated the risks of common adverse events in the co-treatment of malignant-tumor patients with ipilimumab and nivolumab and identified the impacts of different initial administration schemes on the risks of such events. Accordingly, this study provides an important reference for the toxicity of co-treatment with ipilimumab and nivolumab.

## Data Availability Statement

The original contributions presented in the study are included in the article/**Supplementary Material**. Further inquiries can be directed to the corresponding author.

## Author Contributions

XZ, FG, and ZL conceived the study, had full access to all the data in the study, and take responsibility for the integrity of the data and the accuracy of the data analysis. XZ and FG designed the search strategy and discussed with JY, HF, and QX, BG, QY, and KJ performed study selection, data extraction and synthesis. XZ and FG drafted and led on the writing of the manuscript. All the other authors participated in the analysis and interpretation of the data, revised the manuscript critically for important intellectual content and re-drafted some of its section. All the authors read and approved the final version of the manuscript, and agreed to be accountable for all aspects of the work to ensure its accuracy and integrity.

## Conflict of Interest

The authors declare that the research was conducted in the absence of any commercial or financial relationships that could be construed as a potential conflict of interest.

## Publisher’s Note

All claims expressed in this article are solely those of the authors and do not necessarily represent those of their affiliated organizations, or those of the publisher, the editors and the reviewers. Any product that may be evaluated in this article, or claim that may be made by its manufacturer, is not guaranteed or endorsed by the publisher.

## Supplementary Material

Supplementary Figure 1 Forest plot of any adverse events in N1-I3 subgroup.

Supplementary Figure 2 Forest plot of grade 3 or higher adverse events in N1-I3 subgroup.

Supplementary Figure 3 Forest plot of any adverse events in N3-I1 subgroup.

Supplementary Figure 4 Forest plot of grade 3 or higher adverse events in N3-I1 subgroup.

Supplementary Figure 5 Forest plot of treatment-related deaths in N1-I3 subgroup.

Supplementary Figure 6 Forest plot of adverse events leading to discontinuation in N1-I3 subgroup.

Supplementary Figure 7 Forest plot of treatment-related deaths in N3-I1 subgroup.

Supplementary Figure 8 Forest plot of adverse events leading to discontinuation in N3-I1 subgroup.

Supplementary Figure 9 Forest plot of any adverse events in different cancers.

Supplementary Figure 10 Forest plot of grade 3 and higher adverse events in different cancers.

Supplementary Figure 11 Forest plot of any adverse event leading to discontinuation in different cancers.

Supplementary Figure 12 Forest plot of treatment-related deaths in different cancers.
