# Supplementary figures and images for: Risk of Adverse Events in Cancer Patients Receiving Nivolumab With Ipilimumab: A Meta-Analysis
Source: Front Oncol. 2022 Jun 23;12:877434. doi: 10.3389/fonc.2022.877434 (PMC9260026; doi:10.3389/fonc.2022.877434)

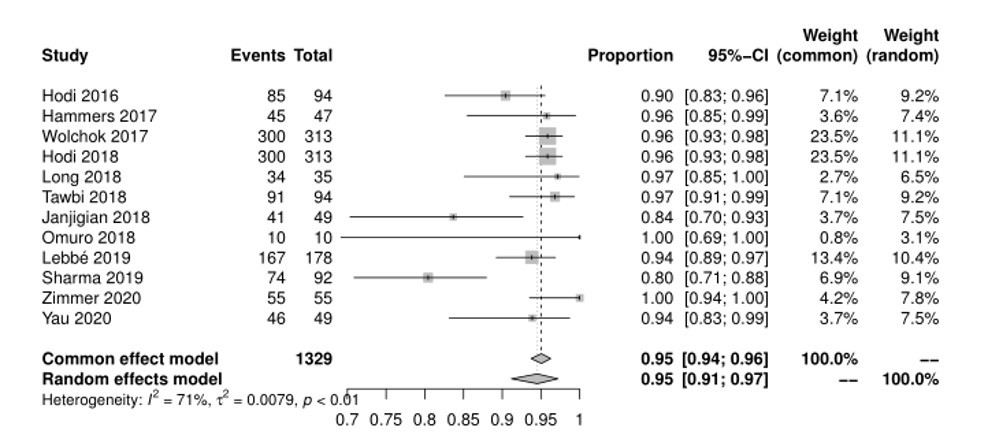

Supplement: Supplementary Figure 1 — Forest plot of any adverse events in N1-I3 subgroup. [file DataSheet_1.zip › Figure S1.jpg]

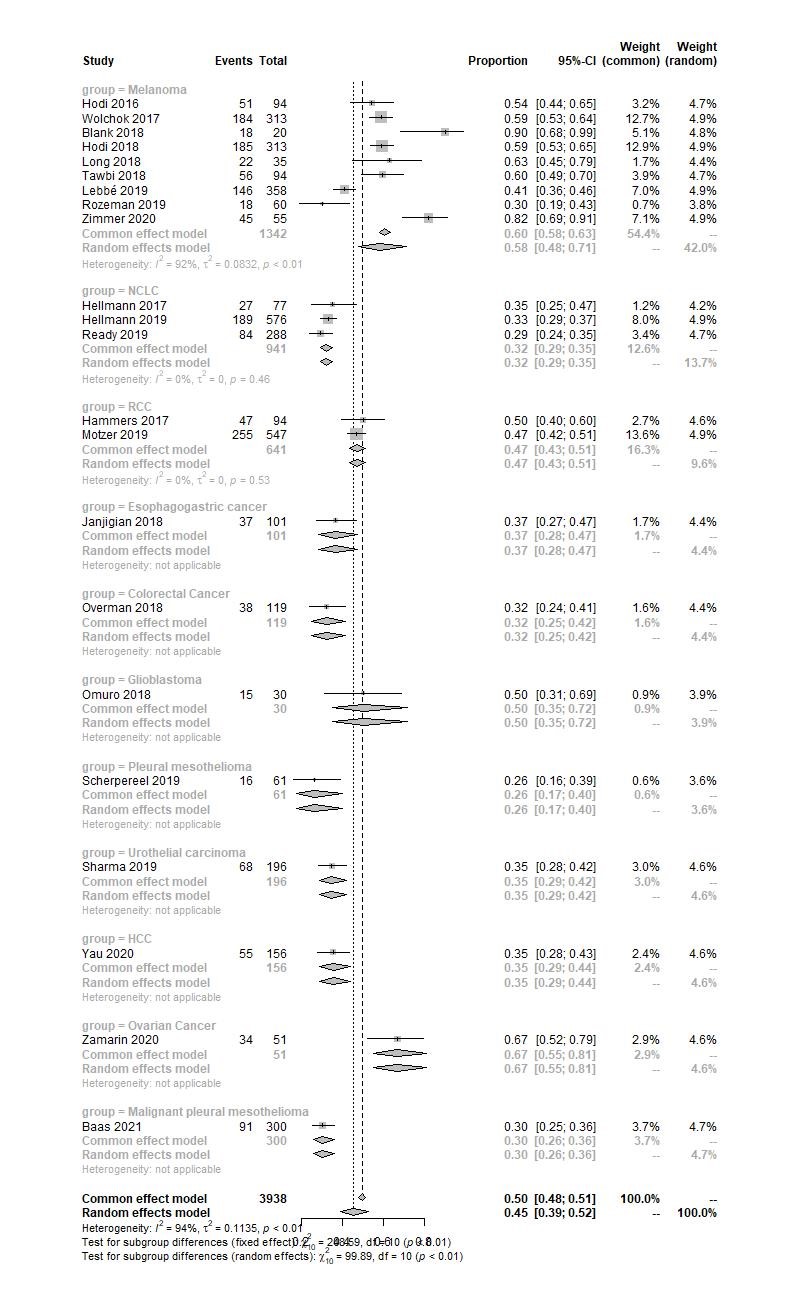

Supplement: Supplementary Figure 1 — Forest plot of any adverse events in N1-I3 subgroup. [file DataSheet_1.zip › Figure S10.jpg]

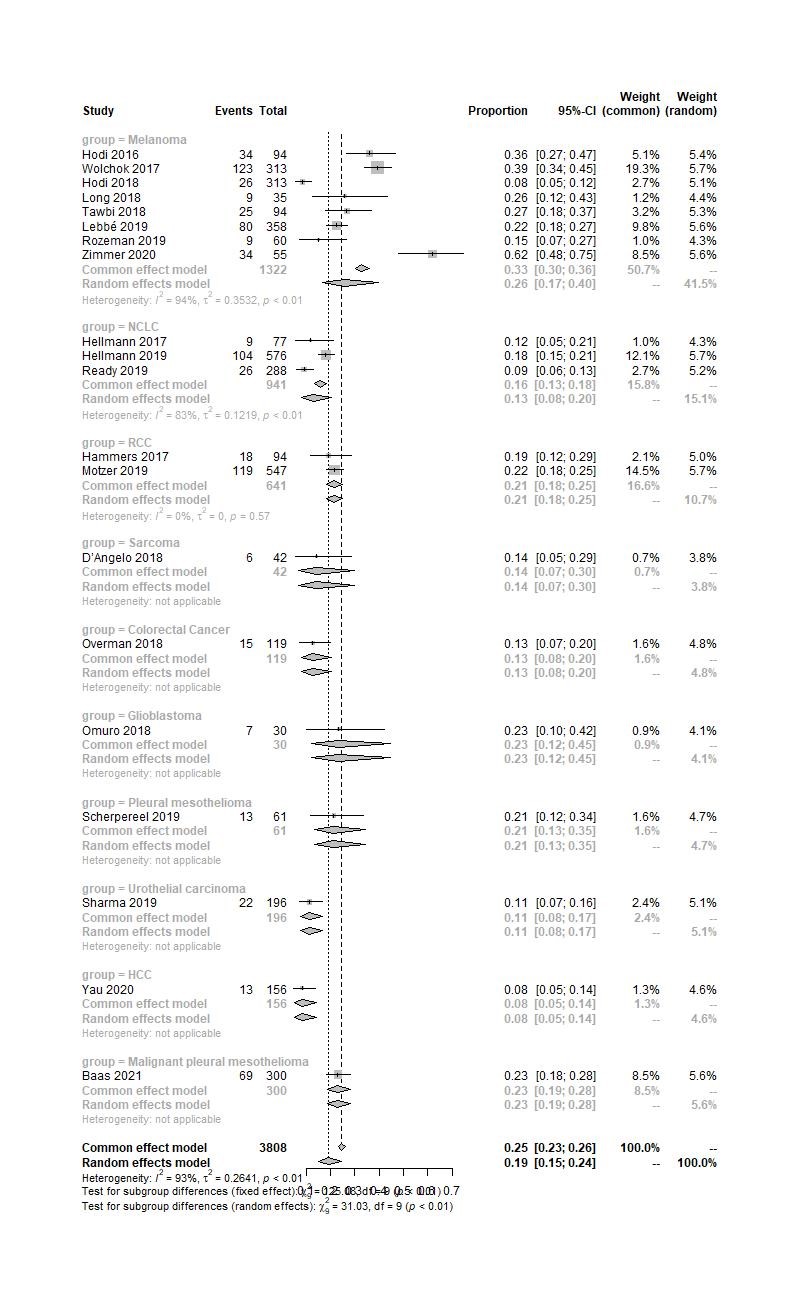

Supplement: Supplementary Figure 1 — Forest plot of any adverse events in N1-I3 subgroup. [file DataSheet_1.zip › Figure S11.jpg]

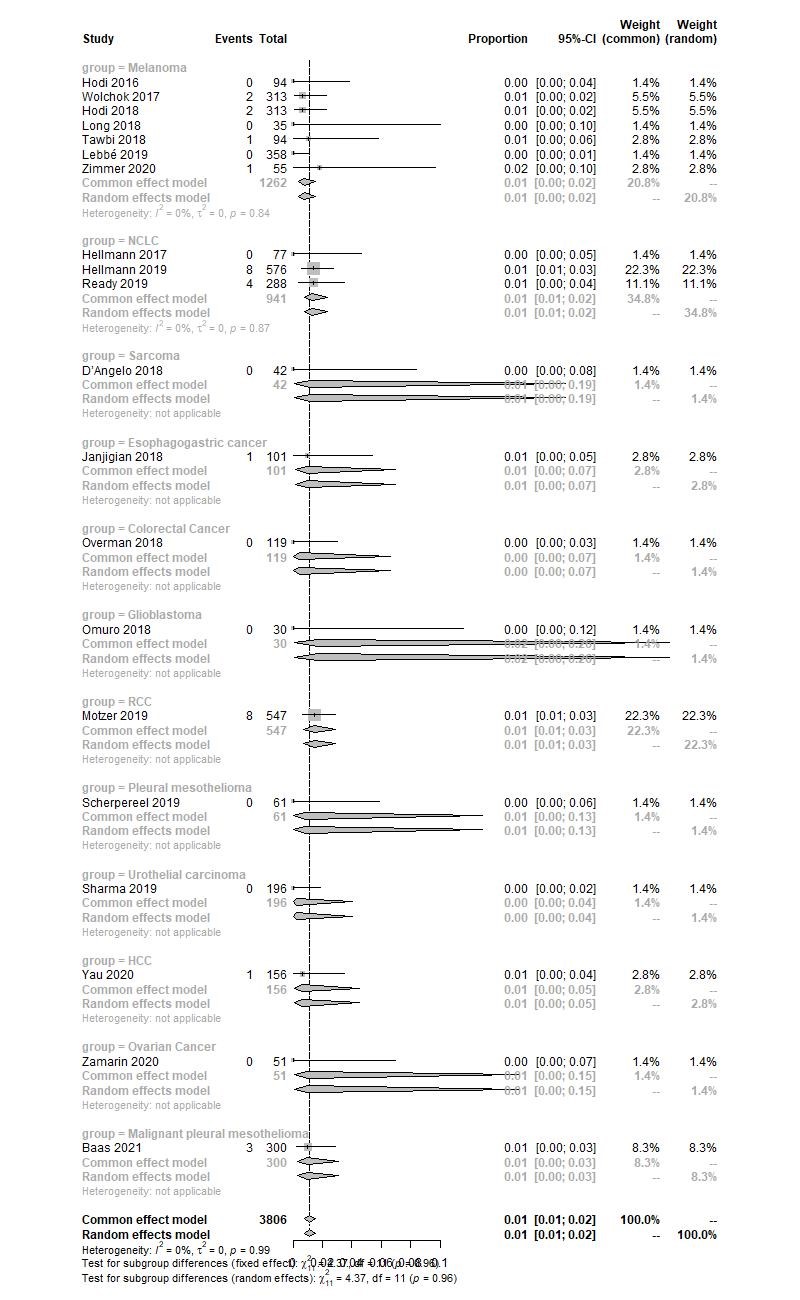

Supplement: Supplementary Figure 1 — Forest plot of any adverse events in N1-I3 subgroup. [file DataSheet_1.zip › Figure S12.jpg]

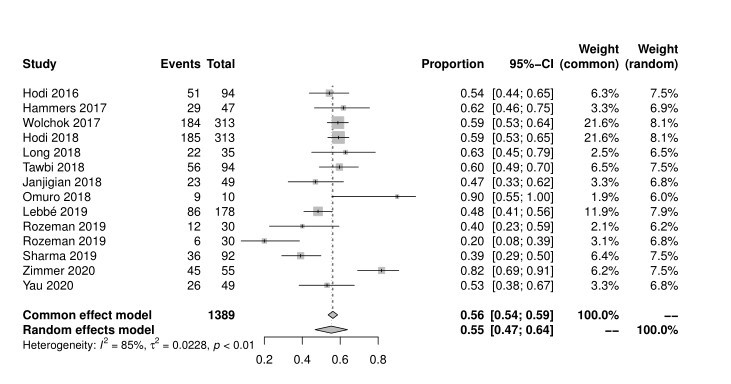

Supplement: Supplementary Figure 1 — Forest plot of any adverse events in N1-I3 subgroup. [file DataSheet_1.zip › Figure S2.jpg]

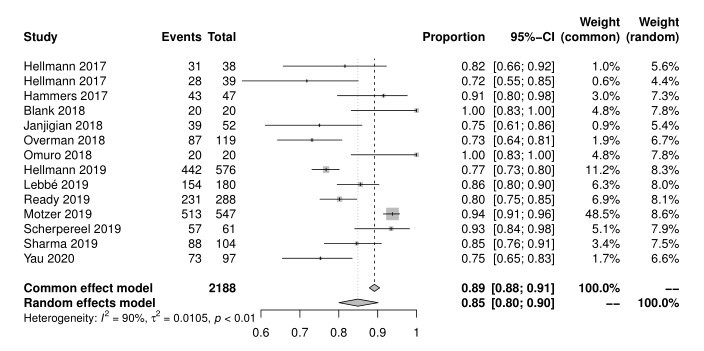

Supplement: Supplementary Figure 1 — Forest plot of any adverse events in N1-I3 subgroup. [file DataSheet_1.zip › Figure S3.jpg]

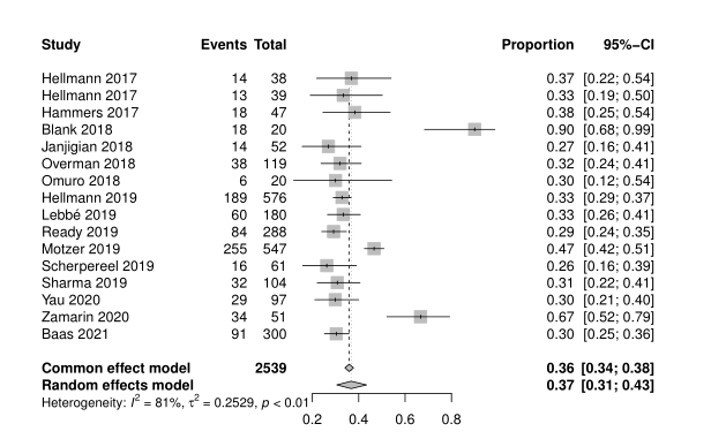

Supplement: Supplementary Figure 1 — Forest plot of any adverse events in N1-I3 subgroup. [file DataSheet_1.zip › Figure S4.jpg]

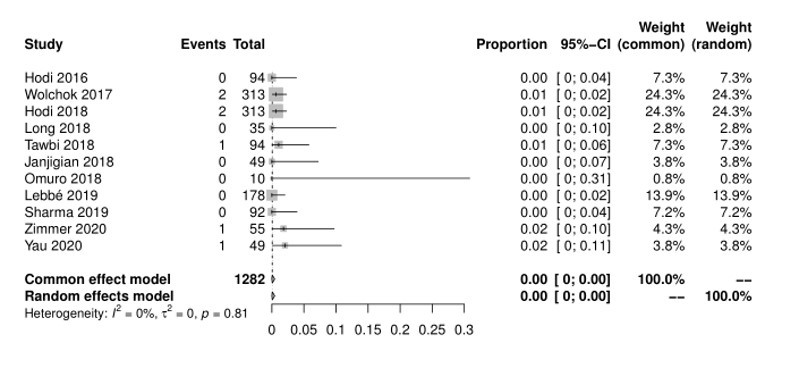

Supplement: Supplementary Figure 1 — Forest plot of any adverse events in N1-I3 subgroup. [file DataSheet_1.zip › Figure S5.jpg]

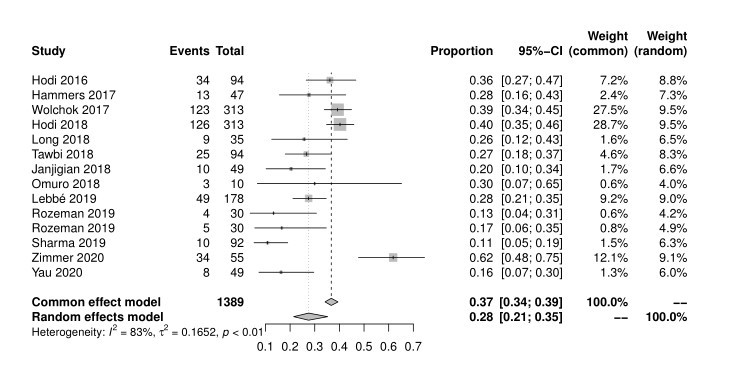

Supplement: Supplementary Figure 1 — Forest plot of any adverse events in N1-I3 subgroup. [file DataSheet_1.zip › Figure S6.jpg]

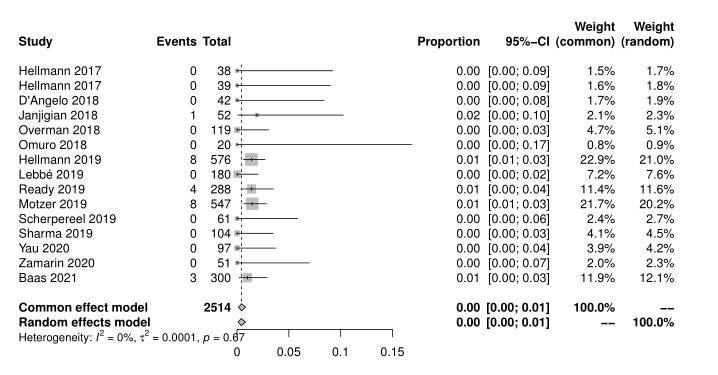

Supplement: Supplementary Figure 1 — Forest plot of any adverse events in N1-I3 subgroup. [file DataSheet_1.zip › Figure S7.jpg]

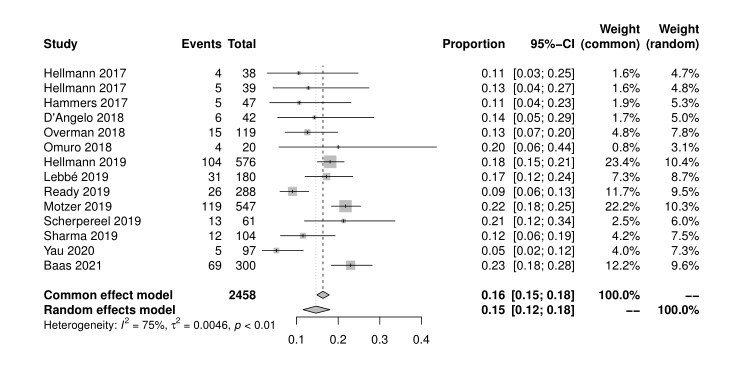

Supplement: Supplementary Figure 1 — Forest plot of any adverse events in N1-I3 subgroup. [file DataSheet_1.zip › Figure S8.jpg]

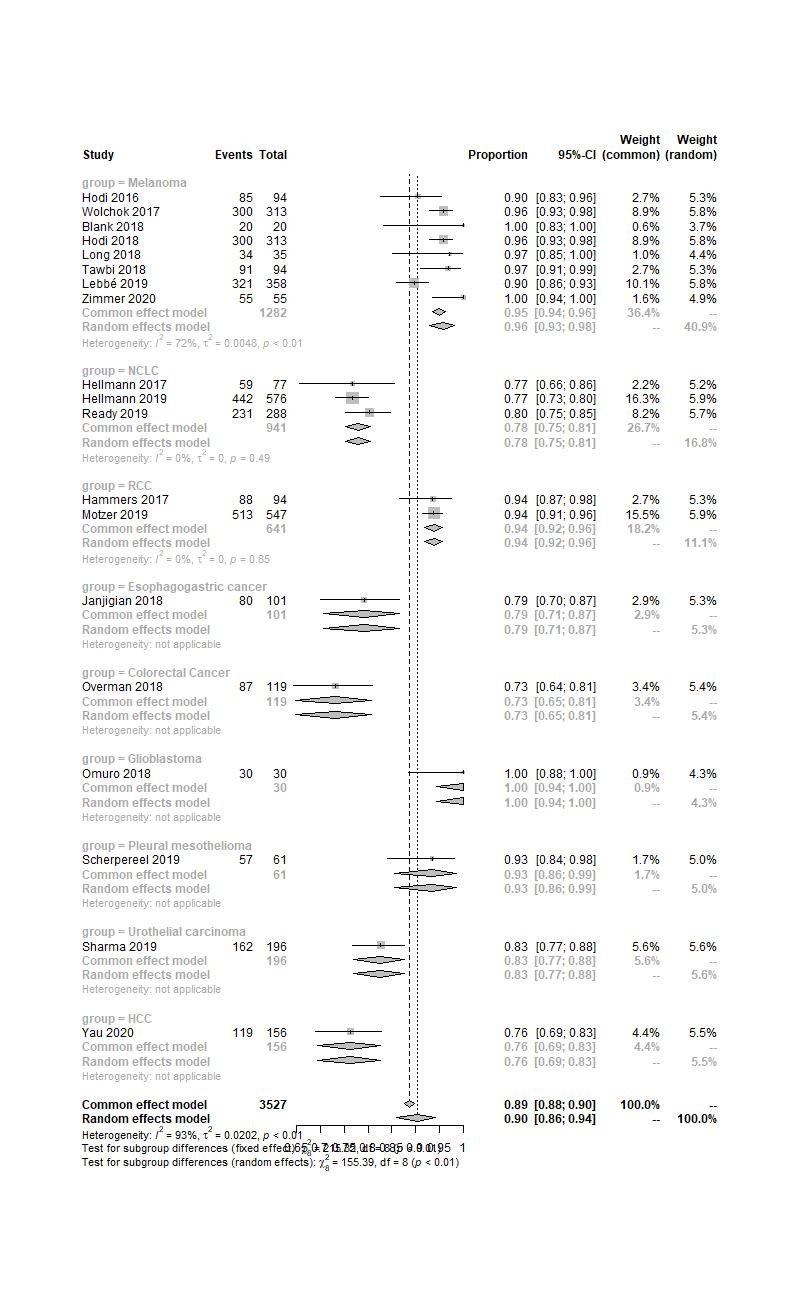

Supplement: Supplementary Figure 1 — Forest plot of any adverse events in N1-I3 subgroup. [file DataSheet_1.zip › Figure S9.jpg]
